# Protein Drug Targets of *Lavandula angustifolia* on treatment of Rat Alzheimer's Disease

**Published:** 2015

**Authors:** Hakimeh Zali, Mona Zamanian-Azodi, Mostafa Rezaei Tavirani, Alireza Akbar-zadeh Baghban

**Affiliations:** a *Faculty of Paramedical Sciences, Shahid Beheshti University of Medical Sciences, Tehran, Iran.*; b *Proteomics Research Center, Faculty of Paramedical Sciences, Shahid Beheshti University of Medical Sciences, Tehran, Iran. *; c *Rehabilitation Faculty, Shahid Beheshti University of Medical Sciences, Tehran, Iran.*

**Keywords:** Alzheimer's disease, *Lavandula angustifolia*, Proteomics, Hippocampus, Drug target

## Abstract

Different treatment strategies of Alzheimer's disease (AD) are being studied for treating or slowing the progression of AD. Many pharmaceutically important regulation systems operate through proteins as drug targets. Here, we investigate the drug target proteins in beta-amyloid (Aβ) injected rat hippocampus treated with *Lavandula angustifolia* (LA) by proteomics techniques. The reported study showed that lavender extract (LE) improves the spatial performance in AD animal model by diminishing Aβ production in histopathology of hippocampus, so in this study neuroprotective proteins expressed in Aβ injected rats treated with LE were scrutinized. Rats were divided into three groups including normal, Aβ injected, and Aβ injected that was treated with LE. Protein expression profiles of hippocampus tissue were determined by two-dimensional electrophoresis (2DE) method and dysregulated proteins such as Snca, NF-L, Hspa5, Prdx2, Apoa1, and Atp5a1were identified by MALDI-TOF/TOF. KEGG pathway and gene ontology (GO) categories were used by searching DAVID Bioinformatics Resources. All detected protein spots were used to determine predictedinteractions with other proteins in STRING online database. Different isoforms of important protein, Snca that exhibited neuroprotective effects by anti-apoptotic properties were expressed. NF-L involved in the maintenance of neuronal caliber. Hspa5 likewise Prdx2 displays as anti-apoptotic protein that Prdx2 also involved in the neurotrophic effects. Apoa1 has anti-inflammatory activity and Atp5a1, produces ATP from ADP. To sum up, these proteins as potential drug targets were expressed in hippocampus in response to effective components in LA may have therapeutic properties for the treatment of AD and other neurodegenerative diseases.

## Introduction

Although the principal cause of AD is not fully elucidated but there exist some hypotheses  trying to clarify mechanism of AD such as amyloid hypothesis, tau hypothesis, cholinergic hypothesis (1,2), herpes simplex virus type 1 (3), age-related myelin breakdown (4,5), and oxidative stress  (6,7). However, AD neuropathology is described by loss of neurons and synapses, loss of memory, associated functional decline, and behavioral disturbances (8, 9) that are microscopically visible amyloid plaques and neurofibrillary tangles (10). Toxic aggregated amyloid fibrils are responsible for disrupting the cell's calcium ion homeostasis and inducing programmed cell death (11). Their accumulation in mitochondria and interaction with mitochondrial enzymes lead to release reactive oxygen species, and affecting metabolic pathways (12). Activated P53 and elevated TNF-α also trigger neuronal cell death (13). 

Todays, the most available clinical research in treating AD pathology try to augment of the neurotransmitter acetylcholine (14,15) so these drugs (tacrine, donepezil, rivastigmine and galantamine) have included acetylcholine precursors, muscarinic agonists, nicotinic agonists, and acetylcholinesterase inhibitors (16). Since plant extract with their effective compounds have positive effects on brain cells, herbal remedies are selective medicine that able assist to memory improvement or treatments to delay or prevent different kind of dementias like AD. These herbal components exert protective effect by antioxidant and anti-inflammatory properties such as those seen in Ginkgo biloba apply to protect cell membranes and to regulate neurotransmitter function (17, 18). LA generally known for their multiple pharmacological effects such as anticonvulsant, sedative, antispasmodic, analgesic, antioxidant, local anaesthetic activity and recently determined that its ability to clear Aβ from rat AD hippocampus (19-24). So it can act as new drug for AD disease.

Many of the pharmaceutically important regulation systems operate through proteins (*i.e*. post-translationally). Major drugs act by binding to proteins. Important drug targets relevant to neurological disorders that have been studied by proteomic technologies include receptors for neurotransmitters, G-protein-coupled receptors and N-methyl-D-aspartate. Other important targets are cell signaling pathways and protein kinases. For instance protein kinase C (PKC) has been implicated in many disorders, such as depression. It was approved decreasing PKC and increasing bcl-2 in the CNS after administrating valproic acid for the treatment of manic-depressive disorders, (25). In chronic lithium administration also seen reduction in the expression of PKC and its substrate, MARCKS, implicate in long-term potentiation. Lithium effects on GSK-3 so lead to neuroprotective/ neurotrophic effects in the brain (26). Nerve growth and repair in AD are associated to design drugs that act as gamma secretase inhibitor, Aβ inhibitor, APOE modification, presenilin gene function (27) or antagonist for cell membrane protein, metabotropic glutamate receptor 5 (mGluR5),that recently reveals by Ji Won Um and his team (28), restoring in memory, learning, and synapse density in AD patients. Therefore multifactorial pathogenesis in AD provided discrete biochemical targets for drug screening and development (29). In addition to novel gene targets associated with AD can be identified by study its interaction with several proteins known to be associated with AD. Abnormal protein folding and aggregation as well reveal critical role in the pathogenesis of neurodegenerative diseases so, comprehending the molecular mechanisms of these abnormality, and their following effects, could therefore help to find therapy targets. So in this study, we seek the some protein targets of protective effect of LA in AD model rat hippocampus that studied by proteomics techniques.

## Experimental

*Materials*

All chemicals used in this study were purchased from Sigma-Aldrich (St. Louis, MO, USA) with exceptions noted. Criterion precast polyacrylamide gels, TGS and XT MES electrophoresis running buffers, Ready Strip™ IPG strips, mineral oil, dithiothreitol (DTT), iodoacetamide (IA), Biolytes, and urea were purchased from Bio-RAD (Hercules, CA, USA). 

*Animals*

Adult male Wistar rats, weighing 250–300 g were housed three to four per cage in a temperature-controlled colony room under light/dark cycle and free access to water and food throughout the experiment. This study was conducted in accordance with the policies stipulated in the Guide for the Care and Use of Laboratory Animals (NIH).

*Experimental procedure*

Rats (n = 30) were randomly allocated to the following groups: (1) sham operation (N; n = 10); (2) Aβ injected group (Aβ; n = 10) and (3) Aβ injected and treated withLE (Aβ+LE; n = 10). For stereotaxic surgery, rats were anesthetized with a combination of ketamine (100 mg/Kg*, i.p*.) and xylazine (5 mg/Kg, *i.p*.) and then placed in a Stoelting stereotaxic apparatus (incisor bar –3.3 mm, ear bars positioned symmetrically). The scalp was cleaned with iodine solution and incised on the midline, and a burr hole was drilled through the skull and Aβ 1–40 (Sigma Aldrich, St. Louis, MO, USA) was injected at coordinates of –3.5 mm posterior to bregma, 2 mm lateral to sagittal suture, and 2.8 mm below dura, according to the stereotaxic atlas (30). The animals in control group were treated with the same procedure except that they received distilled water.

Lavender aqueous extract prepared according to Soheili procedure (20). 20 days after establishing AD model, lavender extract (200 mg/Kg) administrated as intraperitoneally injected once per day for 20 consecutive days. The dosage was chosen according to the results of our pilot study and an earlier investigation (31). The sham groups were either injected distilled water. 

*Sample preparation and two-dimensional gel electrophoresis (2DE)*

Fresh hippocampus tissues were snap frozen and kept in liquid nitrogen until use. Hippocampus were washed twice by PBS and 10% Protease Inhibitor then homogenized by pestle in lysis buffer containing 7 M Urea, 2 M Thiourea, 4% CHAPS(3-(3-Cholamidopropyl) dimethylammonio)-1-propanesulfonic acid), 20 mM Tris, 10 mM DTT (Dithiothreitol), and Protease Inhibitor (one tablet in 2 mL lysis buffer). Homogenates were sonicated ten times on ice for 10 s and left for one hour at room temperature. Lysates were centrifuged at 20000×g for 30 min at 12 °C. Protein concentrations were determined by Bradford assay. 1200 µg from each sample was resuspended in rehydration buffer containing 8 M urea, 4% CHAPS, 0.2% Ampholyte, 50 mM DTT for 16 h and then loaded onto 11 cm immobilized nonlinear gradient strips (Ph=3-10) (Bio-Rad, Hercules, CA, USA). Strips were focused at 20 °C with the following program: 1000 V for 1 h with linear increase, followed by linear increase to 3000 V, then for 6 h remained on 10,000 V with gradient increase, finally for 2.30 h by linear increase to 1000 in a PROTEAN®i12TM IEF Cell (Bio-Rad). The strips were reduced in equilibration buffer containing 20% glycerol, 2% SDS (Sodium Dodecyl Sulfate), 6 M urea, 50 mMTris-HCl and 2% DTT for 20 min and subsequently alkylated in the same buffer containing 2.5% iodoacetamide instead of DTT for 20 min. The IPG (immobilized pH gradient) strips were placed on 12% polyacrylamide gels and electrophoresed initially for 30 min at 16 mA/gel and then 6hr at 24 mA/gel using the protein Xi-II cell (BioRad laboratories)(32). Resulting gels were stained with Coomassie Brilliant Blue (33).

**Table 1:** Differences related to statistically significant (P < 0.05) hippocampus proteome changes in N, Aβ and Aβ+ LE obtained by 2-DE gels.

**Accession No. Swiss-Prot**	**name**	**Protein name**	**Protein expresion**			**Anova (p) **	**Protein abundance ratio **	**Biological process**	**Molecular function**	**Cellular component**	**KEGG_PATHWAY**
			N	Aβ	Aβ+LE						
P37378	SYU1 RAT	Alpha-synuclein, forms 1 and 3	1.22E+04	1.17E+04	2.46E+04	4.64E-08	2.1	Cellular response to stress, microglial cell activation, regulation of receptor recycling, regulation of protein amino acid phosphorylation, positive regulation of neurotransmitter secretion, synaptic transmission, dopaminergic, myeloid leukocyte activation, oxidative phosphorylation, catecholamine metabolic process, fatty acid metabolic process, cellular ion homeostasis,	Fatty acid binding, phospholipid binding, cytoskeletal protein binding, protein transmembrane transporter activity, protein transporter activity,P-P-bond-hydrolysis-driven transmembrane transporter activity, tubulin binding, Hsp70 protein binding, dynein binding, tau protein binding, arachidonic acid binding,	Cell fraction, mitochondrion, microsome, cytosol, cytoskeleton, synaptic vesicle, clathrin-coated vesicle, axon,	Alzheimer's disease, Parkinson's disease
P37377	SYU1 RAT	Alpha-synuclein, forms 1 and 3	1876.088	2471.546	4146.322	3.10E-08	2.2	-	-	-	-
P06761	GR78 RAT	Heat shock protein 5	2052.751	1565.22	3879.16	1.70E-07	2.5	Cellular response to stress, anti-apoptosis, ER overload response,	Nucleotide binding,enzyme inhibitor activity, endopeptidase inhibitor activity, caspase inhibitor activity	Extracellular region, endoplasmic reticulum	Antigen processing and presentation, Prion diseases
P35704	TDX1 RAT	Thioredoxin peroxidase 1 (thiol-specific antioxidant protein)	6265.223	4560.234	1.26E+04	5.45E-06	2.8	Cellular response to stress, MAPKKK cascade, activation of MAPK activity, response to reactive oxygen species, cell activation, response to molecule of bacterial origin, immune effector process,	Peroxidase activity, thioredoxin peroxidase activity, antioxidant activity, oxidoreductase activity, acting on peroxide as acceptor,peroxiredoxin activity,	Mitochondrion, cytosol,	
O09054	APOA1_RAT	Apolipoprotein A-I	874.559	1092.466	698.949	0.001	1.6	Cellular response to stress, cell morphogenesis	Beta-amyloid binding, enzyme inhibitor activity, lipid transporter activity	Extracellular region, extracellular space, endoplasmic reticulum	PPAR signaling pathway,
P15999	ATPA RAT	ATP synthase alpha chain, mitochondrial precursor (EC 3.6.1.34) (fragment)	1365.504	2021.744	1577.425	9.54E-05	1.5	Regulation of endothelial cell proliferation, oxidative phosphorylation,	ATPase, F1/V1/A1 complex, alpha/beta subunit, nucleotide-binding domain,	Mitochondrial proton-transporting ATP synthase complex, catalytic core F(1)	Oxidative phosphorylation, Alzheimer's disease, Parkinson's disease, Huntington's disease,
P19527	NFL RAT	Neurofilament triplet L protein (68 kDa neurofilament protein) (NF-L)	603.587	656.358	2204.352	1.82E-08	3.7	Cellular response to stress, microtubule cytoskeleton organization, cell morphogenesis, protein complex assembly,	Structural molecule activity, enzyme binding, phospholipase binding,	Cytosol, cytoskeleton, intermediate filament, neurofilament, axon, TSC1-TSC2 complex, cell projection, neuron projection	Amyotrophic lateral sclerosis (ALS),

*Protein identification by MALDI-TOF/TOF*

In-gel protein digestion was performed according to Zhou *et al.* with minor modifications (34). The data search was conducted on GPS Explorer (Version 3.6, AB SCIEX) using the search engine Mascot (Version 2.2, Matrix Science, London, UK), and the International Protein Index (IPI) rat database (vision 3.64, 39871sequences, http://www.ebi.ac.uk/IPI) was used for peptide and protein identification. General protein identification was based on two or more peptides whose ion scores surpassed the statistical threshold (p<0.05). 

*Bioinformatics and statistical analysis*

Scanned 2DE gels were analyzed by using Non-linear Progenesis Same Spot software to compare gels together and compare the spots in one statement in gels and get the density of same spot in each of gel. To detect significant differences between the experimental groups, analysis of variance (ANOVAs) were used. A p-value <0.05 was considered to be statistically significant. Statistics were presented as means ± SE.

The identified proteins were then matched to specific processes or functions by searching the GO in DAVID Bioinformatics Resources 6.7 (the Database for Annotation, Visualization, and Integrated Discovery) (35), “http://david.abcc.ncifcrf.gov/”, a comprehensive set of functional annotation tools for understanding the biological meaning behind genes.

Identified proteins were used to determine predicted interactions with other proteins. This functional protein association network for the each entry was obtained by searching the STRING online database (http://string-db.org). 

## Results

**Figure 1:**
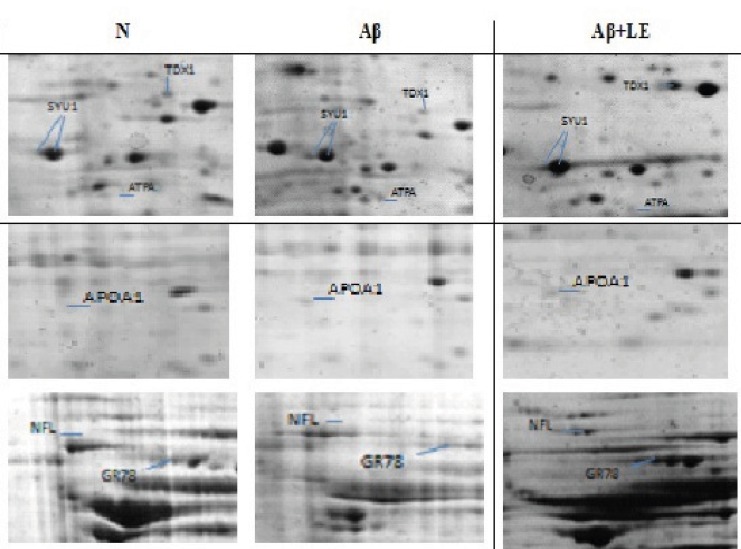
Representative part of two-dimensional gel maps of proteins (N, Aβ and Aβ + LE) that MS-identified spots showing significant alterations in experimental groups are displayed in representative gels with corresponding identities.

To explore the molecular mechanism underlying the beneficial effect of lavender aqueous extract on clearance of Aβ from hippocampus of AD rat model, 2DE-based proteomics was utilized and the differentially expressed proteins in the N, Aβ and Aβ+ LE groups were analyzed. As shown in Figure 1, Snca, Hspa5, Prdx2, Apoa1, Atp5a1, and NF-L were detected by Coomassie Brilliant Blue in three 2DE maps validated by MALDI-TOF/TOF analysis. The MS data were queried using the search algorithm GPS 3.6 (mascot 2.2) against the IPI rat database. Proteins were identified based on a number of criteria including their expressions in three groups, alteration ratio, GO-discovered categories and KEGG pathways illustrated in Table 1. All of proteins are up-regulated by LE except APOA1 and ATPA that inhibited by LE and over-expressed in Aβgroup. The GO-discovered categories to find biological process, molecular function and cellular components used DAVID analysis. For determined KEGG pathways also used DAVID analysis which the results depicted relationship between some proteins and neurodegenerative disease pathways. 

*Results of protein association network*

**Figure 2:**
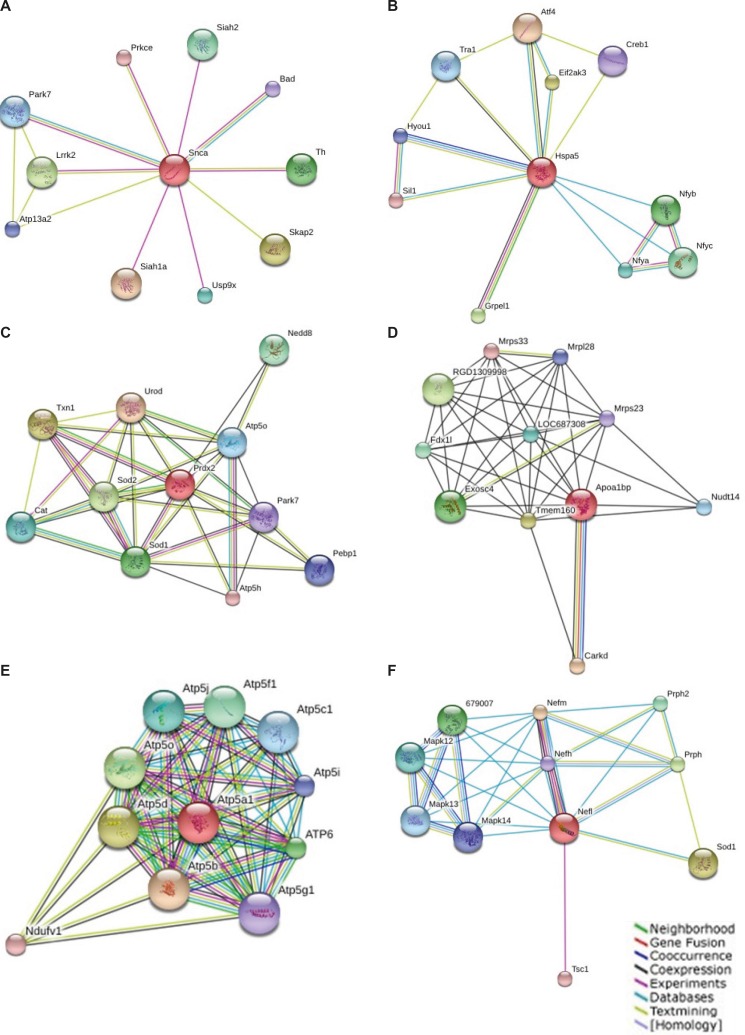
Known and predicted interactions of Snca (a), Hspa5 (b), Prdx2(c), Apoa1bp(d), Atp5a1(e) and NF-L(f) with other proteins. This functional protein association network was obtained by searching the STRING online database (http://string-db.org).

Known and predicted interactions of detected proteins in GO classification with other proteins were obtained by searching the STRING online database and exhibited in Figure 2. This functional protein association network for the entry “Snca” binds directly to proteins such as Siah1a, Skap2, Lrrk2, Th, Siah2, Usp9x, Park7, Atp13a2 and Bad. Hspa5 binds directly to proteins Atf4, Eif2ak3, Grpel1, Nfyb, Nfyc, Nfya, Tra1, Hyou1, Creb1, and Sil1. Prdx2 binds to proteins Urod, Txn1, Sod2, Sod1, Nedd8, Cat, Atp5o, Pebp1, Park7 andAtp5h. Apoa1bp binds Carkd, Tmem160, RGD1309998, Exosc4, Fdx1l, LOC687308, Nudt14, Mrpl28, Mrps23 and Mrps33. Atp5a1 bind to Atp5b, Atp5d, Atp5o, ATP6, Atp5f1, Atp5j, Atp5c1, Atp5i, Ndufv1 and Atp5g1. NF-L binds to Nefm, Sod1, Prph, 679007, Prph2, Mapk12, Mapk13, Mapk14, Nefh and Tsc1.

## Discussion

AD has been well known as a proteopathy disease which caused by formation of Aβ plaqueand tau tangles in the brain (36). In addition to Aβ plaque, there are toxic oligomers of Aβ that bind to neuronal surface receptors. These interactions play important roles in loss of neurons connections (37, 38). On the other hand, tauopathy fragmentize the neuron's transport system (39), so the researchers are interested to reduction of amyloid levels, prevention of amyloid aggregation/toxicity and tau phosphorylation/aggregation. Since several different proteins, such as APP, APOE, BACE (Aβ cleaving enzyme), PS1/2, secretases, and tau play important roles in the pathology of AD, Therefore, inhibition of BACE, PS-1 and g-secretase are important activities in treatment of AD. A number of strategies such as acetylcholinesterase inhibitors, anti-oxidants, anti-inflammatory agents, hormone therapy, cholesterol-lowering agents and vaccination, are being investigated for treating or slowing the progression of AD (1-6). Today, herbal treatments as mainly alternative medicine for Alzheimer's are widely expanded. Here, neuroprotective mechanism of LE wasinvestigated by proteomics approach. Previously determined that such protective effect might be related to antiglutamatergic, calcium channel blocking and antioxidant activity (24, 40, 41). These pharmacologic activities of lavender linked to presence of a lot of monoterpenes (especially linalyl acetate, linalool) (22, 42). Proteomics act as effective target discovery system that applied to rapid identification of proteins, which are likely to be targets for therapeutic development. As represented in Figure 1, the analysis of hippocampal proteome determined some proteins with different expression level in three groups (N, Aβ and Aβ+LE).

These proteins belong to the biological process correlate neuroprotective effect of LA tomake removing Aβ plaques or restructuring AD neurons after treatment with LE. These proteins included Snca, Hspa, Prdx2, Apoa1, Atp5a1 and NF-L which comprehensively are explained in table 1. Protein–protein interaction as a powerful proteomic activity is applied for interpretation of interactions of these proteins to each other's. Beside on, the brain function likewise other organs can be explained by protein complexes in different terms such as the number, type and location. Protein networks analysis by proteomics tools can help to description of neurobiological phenomena (43). In addition to, co-expression patterns of genes displaying as gene set, protein complex or part of a functionally related pathway, so in this study proteins are studied in network models for more resolution.

Alpha-synuclein (Snca), the most important protein in this study, is aprotein whose accumulation is common to many neurodegenerative diseases (44), and it is non-Aβ component in AD amyloid that modulates the APP mRNA. This protein is found only in brain (hippocampus, brain stem and cortex). Snca is tau protein binding and can interact significantly with tubulin (45, 46), and it is also acts as a molecular chaperone in configuration of SNARE complexes (47, 48). Several Yeast genes in lipid metabolism display in Snca toxicity (49) in contrary its possible antioxidant activity (50). Since Snca specifically expressed in neuronal cell bodies and synapses, it may be involved in the regulation of dopamine release and transport phenomena, so that has been reported to accumulate abnormally in AD, PD and inflammatory demyelinating disease (44). Additionally, mutations in Snca were found to be associated with rare familial cases of early-onset of PD (51, 52). Amongst many versatile functions of Snca, it has been mainly described to beinvolved in the regulation of membrane stability and/or turnover and display in synapse, axon, synaptosome, clathrin-coated vesicle, and cellular response to oxidative stress. Binding proteins (Figure 2a), like Bad, have been identified as Snca target (35).Bad (Bcl2 antagonist of cell death) that responses to reactive oxygen species acts in different pathways such as ErbB signaling pathway and apoptosis, which have been extensively described in the context of AD. In this context, interactions with PD associated proteins like leucine-rich repeat kinase 2 (Lrrk2), Protein DJ-1 (Prkce) and Tyrosine3-monooxygenase (Th) may lead in neuron development and differentiation associated with lavender treatment (53, 54). In this study, additional isoforms of Snca seem to exist with different expression while previously specified increasing level of Snca in AD (55). Snca levels were significantly reduced in aged condition in all structures except hippocampus while up-regulated in presynaptic terminals during synaptic rearrangement (56). However, here, up-regulation of several isoforms of Snca was detected in treated with LE sample. Based on conflicting evidences, Snca is associated with a non-apoptotic de novo expression after axotomy of adult rat that shown slow form of neurodegeneration in facial motor neurons (57). Besides, pro-apoptotic function in AD pathogenesis, de novo over-expression of Snca in hippocampus neuron after treatment with LA have a number of its neuroprotective effects such as tau substitution act in cellular transport, cellular localization,vesicle trafficking, membrane stability, cooperation with clathrin and amphiphysin to increase endocytosis, regulation of neurotransmitter transport, membrane depolarization, positive regulation of transmission of nerve impulse, regulation of post synaptic membrane potential, and finally act as oxidoreductase and antioxidant enzyme. 

 Heat shock protein 5 (Hspa5) belongs to the heat shock protein 70 family and displays in protein complex assembly inside the ER (58). It was determined regulatory role of HSPA5 in the stress response (59). It also is anti-apoptotic protein display in prion diseases (Figure 2b) related to proteins such as Eif2ak3 and Creb1. Biological process of Eif2ak3 is regulation of nervous system development and demonstrates in AD, whereas Creb1 be involved in the neuron differentiation and neuron projection development and display in HD (35). In this study, Hspa5 had 2.5 fold more expressionin the presence of lavender while in AD has even lower expression than normal hippocampus. Hspa5 as a heat shock protein implies its neuroprotective function that may be relate to anti-apoptotic protection of LA.

Mitochondrial membrane ATP synthase (Atp5a1) is a protein in respiratory chain deal with generation of ATP from ADP (35), it binds to Atp5b, Atp5d,Atp5o, ATP6, Atp5f1, Atp5j, Atp5c1, Atp5i, Ndufv1, Atp5g1(Figure 2c). All of interacted proteins except Atp5i are composed of pathways like oxidative phosphorylation, AD, PD, HD (35). Beforehand Tsuji T *et al.* (60) determined decreasing amount of Atp5a1 in AD, but in our study, increased expression of Atp5a1 in AD group than the two other groups was seen.

Neurofilament light polypeptide (NF-L) is a member of the actin proteinand intermediate filament (IF). IFs are primordial components of cytoskeleton and nuclear envelope. It seems that level of IF gene expression directly controls axonal diameter, which in turn controls how fast electrical signals travel down the axon. IFs are subdivided into three major subgroups IF- L, M, and H which are involved in the maintenance of neuronal caliber (61-63). NF-L (Figure 2d) binds to Mapk12 (mitogen-activated protein kinase 12), Mapk13, Mapk14, Nefh (neurofilament), Nefm, Prph (peripherin) and Prph2 which are involved in pathway associated to Amyotrophic Lateral Sclerosis (ALS). Sod1, superoxide dismutase 1 that is related to ALS is composed to HD and Prion diseases (35). Increment of NF-L level was specified in AD (64), in this study LA lead to 3.7 fold more expression than the other two groups. This effect may be related to microtubule cytoskeleton organization, regulation of neurogenesis and prevents, or reduces the frequency, rate orextent of cell death by apoptotic process (35). 

Thioredoxin peroxidase 1 (Prdx2, TDX1) is an antioxidant protein involvedin cellular redox regulation. By reducing peroxides through thioredoxin system may act to get rid of peroxides generated during metabolism. It might also regulate the intracellular concentrations of H_2_O_2_ or involved in the neurotrophic process (65). It binds to proteins Atp5h and Atp5o (Figure 2e) that are in oxidative phosphorylation, AD, PD, HD, Park7 (peroxiredoxin activity) in PD, Cat in ALS, Sod1 in ALS, HD and Priondiseases and Sod2 in HD (35). It is reported that its expression increasesin AD (66) but here we found down regulated in AD than the two other groups and LE causes increasing Prdx2 expression to 2.8 fold. 

Apolipoprotein A-I (ApoA-I), the major protein of high-density lipoprotein(HDL), belongs to the apolipoprotein A1/A4/E family takes part in the cholesterol reverse transport from tissues to the liver for excretion. It also exhibits its anti-inflammatory activity in inflammatory responses. As renal inflammation it plays an important role in ischemia/reperfusion (I/R) injury of the kidney. ApoA-I improved renal function by decreasing I/R-induced inflammatory responses such as inhibition release of inflammatory cytokines and neutrophil infiltration and activation (67). It binds Carkd, Tmem160, RGD1309998, Exosc4, Fdx1l, LOC687308, Nudt14, Mrpl28, Mrps23 and Mrps33, (Figure 2f) that all of them are located in mitochondria (35). Previous investigation assigned decreasing ApoA-I in AD (68), but here seen increases to 1.6 fold in AD that might be affiliated to its anti-inflammatory activity.

Previous study illustrated the protective effect of aqueous extract of lavender on eliminating Aβ in in trahippocampal Aβ-injected rat model of AD (20, 30), here we evaluated the mechanism of this beneficial effect related to expression some protective proteins such as Snca, Hspa, Prdx2, Apoa1, Atp5a1 and NF-L as protein target for monoterpen agents (rosmarinic acid, caffeic acid, luteolin 7-Oglucoside,methyl carnosoate (69)) in LA that trigger mechanisms such as anti-lipid peroxidation, antiglutamatergic and anti-inflammatory activities (69).

## Conclusion

In this study pharmaceutically important regulation systems of LA that operate through drug target proteins such as Snca, NF-L, Hspa5, Prdx2, Apoa1, and Atp5a1were investigated. Snca, expressed different isoforms, which exhibited neuroprotective effects by anti-apoptotic properties through interaction with Bad, act as chaperone, regulate membrane stability and display in synapse, axon, synaptosome, clathrin-coated vesicle. NF-L involved in the maintenance of neuronal caliber that over-expressed in the presence of LE may relate to microtubule cytoskeleton organization, regulation of neurogenesis and prevents, or reduces the frequency, rate or extent of cell death by apoptotic process. The roles of Hspa5 likewise Prdx2 as like the other anti-apoptotic proteins upon LA treatment were investigated. Prdx2 also involved in the neurotrophic process. Alteration of Apoa1 experssion that hasanti-inflammatory activity in inflammatory responses was discussed indetail. The last proposed target was Atp5a1; it produces ATP from ADP, binds to the proteins that are composed of oxidative phosphorylation pathways. Based on the wide alteration protein expression affected by LA, it can be concluded that LA components may have a therapeutic potential for thetreatment of AD and other neurodegenerative diseases.
